# PW01-037 – Amyloidosis probability depending on MEFV type

**DOI:** 10.1186/1546-0096-11-S1-A90

**Published:** 2013-11-08

**Authors:** A Simonyan, A Ayvazyan, V Vardanyan, L Kozlovskaya, V Rameev

**Affiliations:** 1 yerevan State Medical University, Yerevan, Armenia; 2 Moscow Medical Academy after I.M. Sechenov, Moscow, Russian Federation

## Introduction

Familial Mediterranean fever (FMF) is a hereditary inflammatory disorder. FMF is an autoinflammatory disease caused by mutations in MEFV, a gene which encodes a 781–amino acid protein denoted pyrin. AA-amyloidosis is the main complication of FMF.

## Objectives

To access the relationship between MEFV genotype and occurrence of amyloidosis in patients with Familial Mediterranean Fever (FMF).

## Methods

69 FMF patients (37 with amyloidosis, 32 – without amyloidosis) were investigated. All 69 patient underwent molecular-genetic investigation (PCR method), 9 different mutant combinations of MEFV gene were detected: 3 homozygous - M694V/M694V(AA) in 25 patients, M680I/M680I(CC) - in 4, V726A/V726A(BB) in 1, 5 compound heterozygous - M694V/V726A(AB) in 17, M694V/M680I(AC) in 9, V726A/M680I(BC) in 5, V726A/R761H(BD) in 1, M680I/R761H(CD) in 1, and 1 heterozygous - M694V/U(AU) in 6 patients.

## Results

3 of 9 detected MEFV genotypes (V726A/V726A, V726A/R761H и M680I/R761H) were not revealed in FMF patients with amyloidosis, but in the present investigated group these genotypes were sporadic (3 patients), so that it’s not possible to decline the probability of amyloidosis development in these genotypes unambiguously. The rest 6 genotypes (M694V/M694V, M694V/U, M694V/M680I, M694V/V726A, V726A/M680I, M680I/M680I) were found either in patients with amyloidosis or without. Particularly M694V- genotypes were distributed approximately equally among 2 subgroups of patients. M694V/M694V-genotype appeared to be more frequent in patients with amyloidosis – in 17 of 37 patients (45,9%) in comparison with patients without amyloidosis – in 8 of 32 patients (25%). Nevertheless, detected difference proved to be non-significant (χ^2^=12,51171, p=0,12981).

## Conclusion

Our investigation hadn’t revealed significant relationship between carriage of definite FMF genotype and development of amyloidosis. The absence of genetic relationship between FMF and amyloidosis may be explained by the fact that MEFV and SAA genes are located on different chromosomes – 16 and 11 respectively, and the carriage of the mentioned genes has unlinked character.

## Disclosure of interest

None declared

